# Preparation of Aqueous Solutions with Information on Solids Using Natural Polymers by Solid‐State Mixing

**DOI:** 10.1002/tcr.202500185

**Published:** 2025-12-06

**Authors:** Keita Yamana, Riku Kawasaki, Atsushi Ikeda

**Affiliations:** ^1^ Applied Chemistry Program Graduate School of Advanced Science and Engineering Hiroshima University 1‐4‐1 Kagamiyama Higashi‐Hiroshima Hiroshima 739‐8527 Japan

**Keywords:** aggregations, crystal engineering, fluorescence, host–guest systems, natural polymers

## Abstract

Some organic molecules exhibit multiple properties such as chiral crystal formation, crystal polymorphism, room‐temperature phosphorescence, mechanochromism, and fluorescence detection by molecular recognition only in their solid, self‐aggregated states (crystalline or amorphous states). These functionalities either disappear or converge to a single physical property when these molecules are dispersed in a solvent. To address this limitation, self‐aggregated guest molecules are dissolved in water using natural polymers as solubilizing agents. However, conventional solid–liquid extraction methods such as heating and stirring or ultrasonic irradiation are rendered ineffective in completely dissolving the functional guest molecules in water. These molecules are mixed with natural polymers via grinding or high‐speed vibration milling, followed by extraction with water, to enhance their water solubility while maintaining their functions. These systems are referred to as aqueous solutions with information (properties) on solids.

## Graphical abstract

The solid‐state information, such as chiral crystal formation, crystal polymorphism, mechanochromism, room‐temperature phosphorescence, and fluorescence detection by molecular recognition, is maintained even when the functional guest molecules are solubilized in water using natural polymers.

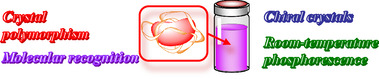

## 1 Introduction

The use of calixarenes and their analogs as host molecules and hydrophobic organic compounds as guest molecules in host–guest chemistry has been extensively studied.^[^ ^1^, ^2^ ^]^ In organic solvents, hydrophobic guest molecules exist in equilibrium between free (uncomplexed) and complexed forms with host molecules. In contrast, hydrophobic guest molecules that are insoluble in water precipitate when not bound to a host molecule in aqueous solutions. Thus, the physical properties of guest molecules in aqueous solutions are entirely determined by those in the host–guest complex.

**Scheme 1:**
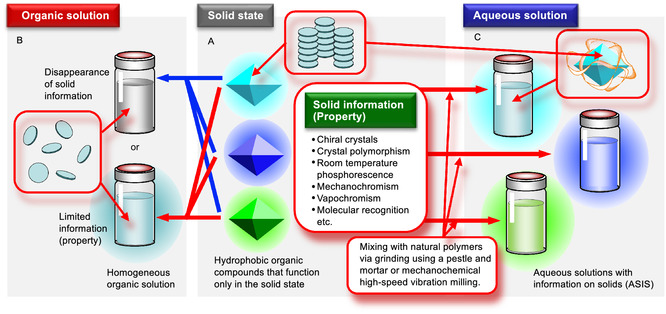
A) Some organic molecules exhibit solid‐state information. B) When dispersed in organic solvents, the information (properties) either disappeared [e.g., aggregation‐induced emission (AIE), chirality in achiral molecular crystals, and room temperature phosphorescence (RTP)] or became limited (e.g., crystal polymorphism). C) An aqueous solution containing a natural polymer–guest molecule complex, prepared by grinding using a pestle and mortar or by mechanochemical high‐speed vibration milling (HSVM), retained the solid‐state information.

Cyclodextrins, which are macrocyclic oligosaccharides, can encapsulate guest molecules into their cavities in a 1:1 or 2:1 host–guest ratio, enabling the dispersion of guest molecules in water.^[^ ^3^, ^4^, ^5^, ^6^, ^7^ ^]^ For instance, the ultraviolet–visible (UV–vis) absorption spectrum of [60]fullerene sandwiched between two γ‐cyclodextrins is almost identical to that of cyclohexane.^[^ ^8^, ^9^ ^]^ Thus, the guest molecule in cyclodextrin has almost the same physical properties as that dissolved in an organic solvent. However, owing to their small particle size, cyclodextrin‐based host–guest complexes cannot achieve the enhanced permeability and retention (EPR) effect, which refers to the accumulation of macromolecules, aggregates, or nanoparticles in tumor tissues. This accumulation occurs because of the increased vascular permeability caused by angiogenesis and reduced clearance from the lack of functional lymphatic drainage in solid tumors.^[^ ^10^, ^11^, ^12^ ^]^ Therefore, complexes that utilize micelles or water‐soluble polymers as solubilizing agents may be clinically applicable, since they accumulate in tumors via the EPR effect.^[^ ^12^, ^13^, ^14^ ^]^ In contrast, hydrophobic guest molecules often self‐aggregate when dissolved in micelles or water‐soluble polymers.^[^ ^15^, ^16^, ^17^ ^]^ Although complexation with water‐soluble polymers improves the stability of hydrophobic guest molecules against light irradiation, self‐aggregated fluorescent molecules, such as porphyrin derivatives and analogs, become self‐quenched and lose their photodynamic activity, which hinders the application of such organic compounds as functional materials. Nevertheless, organic crystallography has revealed their diverse physical properties in the solid state, such as chiral crystals,^[^ ^18^, ^19^ ^]^ crystal polymorphism,^[^ ^20^, ^21^ ^]^ room‐temperature phosphorescence,^[^ ^22^, ^23^ ^]^ mechanochromism,^[^ ^24^, ^25^ ^]^ and vapochromism (**Scheme** **1**A).^[^ ^26^, ^27^ ^]^ In many cases, this information (properties) that arises from solid‐state self‐aggregation is lost when the materials are dispersed in organic solvents (Scheme 1B).

Therefore, we hypothesized that such self‐aggregated guest molecules could be made water‐soluble while retaining their solid‐state properties. To this end, aqueous solutions with information on solids (ASIS) were prepared herein (Scheme 1C). As a result, we showed that guest molecules could be dissolved in water while retaining their solid‐state physical properties. Compared with their solid‐state counterparts, ASIS is easier to handle and can be readily applied to substrates while maintaining various solid‐state properties. Furthermore, their aqueous nature makes them particularly promising for applications in biological systems. To enable solubilization, we employed natural polymers, such as polysaccharides and polypeptides. Many of these polymers are commercially available, exhibit high biocompatibility, and are well‐suited for biomedical use. Additionally, the use of these naturally derived materials promotes environmental sustainability.

## 2 Water Solubilization of Hydrophobic Guest Molecules Using Natural Polymers

Our ASIS system was functionalized by incorporating hydrophobic guest molecules into complexes, using natural polymers as solubilizing agents. However, as this process requires solid‐state mixing, we will begin by introducing the solid‐state mixing procedure.

**Scheme 2:**
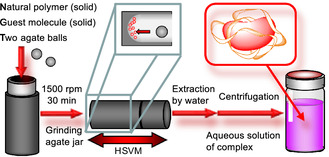
Preparation of an aqueous solution containing a natural polymer–guest molecule complex via mechanochemical high‐speed vibration milling. Reproduced with permission.^[^ ^110^ ^]^ Copyright 2023, Wiley‐VCH.

Solid–liquid extraction is employed for the water solubilization of guest molecules using polysaccharides or polypeptides as solubilizing agents for hydrophobic molecules. These polymers are first dissolved in water and solid hydrophobic guest molecules are added to the solution. This solution is heated and stirred or subjected to ultrasonic irradiation to increase the extraction rate. However, this method offers very low extraction yield even after the treatment exceeds 24 h. Akiyoshi et al. addressed this issue by chemically modifying polysaccharides with hydrophobic moieties such as cholesterol to synthesize self‐assembled nanogels that encapsulated hydrophobic guest molecules mainly via hydrophobic interaction.^[^ ^28^, ^29^, ^30^, ^31^ ^]^ Since then, various types of nanogels have been synthesized.^[^ ^32^, ^33^, ^34^ ^]^ Herein, we demonstrated that various hydrophobic compounds can be made water‐soluble via mechanochemical high‐speed vibration milling (HSVM)—a type of ball mill—without requiring chemical modification of these bio‐based polymers (**Scheme** **2**).

**Figure 1:**
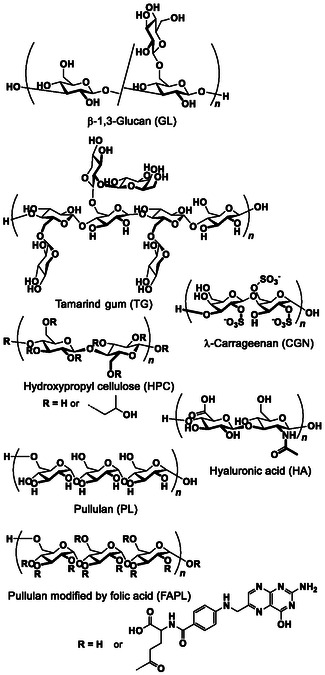
Chemical structures of polysaccharides used as solubilizing agents.

**Figure 2:**
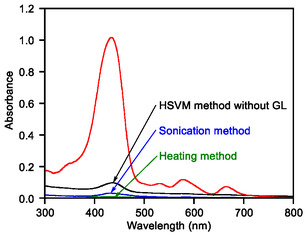
UV–vis absorption spectra of porphyrin derivative **10** prepared using the HSVM method (black) and of the β‐(1,3‐1,6)‐D‐glucan (GL)–**10** complex obtained using the HSVM (red), sonication (blue), and heating (green) approaches. In each method, the solutions were prepared using GL (10.0 mg), **10** (2.0 μmol), and water (2.0 mL). All absorption spectra were measured in H_2_O at 25 °C using a cell with a path length of 1 mm. Reproduced with permission.^[^ ^35^ ^]^ Copyright 2021, Wiley‐VCH.

Cyclodextrins and polysaccharides are composed of glucose; however, they have significantly different properties besides the state of guest molecules—as mentioned in the Introduction section—particularly due to humidity, as will be discussed later. Komatsu et al. prepared an aqueous solution of a γ‐cyclodextrin–C_60_ complex via HSVM; its concentration was ≈9–18 times higher than the concentrations of complexes prepared using conventional methods, such as solid–liquid extraction and classical ball‐milling.^[^ ^9^ ^]^ Furthermore, the HSVM method facilitated a much higher solubilization of a complex comprising β‐(1,3‐1,6)‐D‐glucan (GL, **Figure** **1**) and a porphyrin derivative (**10**), GL–**10**, than conventional solid–liquid extraction methods, such as heating and stirring or ultrasonic irradiation. This finding was supported by the absorbance of **10** (**Figure** **2**).^[^ ^35^ ^]^ In HSVM, host molecules (or solubilizing agents) such as cyclodextrins and natural polymers are mixed with solid hydrophobic guest molecules and extracted using water. However, during the rainy season in Japan, the water‐based extraction of γ‐cyclodextrin–C_60_ complex is challenging via HSVM. Therefore, γ‐cyclodextrin must be dried under low pressures (<20 Pa) and high temperatures (>80 °C) before use. Alternatively, a mortar and pestle can be used for extracting C_60_; however, this method offers very low extraction yields (unpublished data) because γ‐cyclodextrin absorbs atmospheric moisture during long‐term grinding. Such issues are seldom observed with polysaccharides. These findings collectively indicate that macrocyclic cyclodextrin changes its structure, which was unsuitable for the inclusion of C_60_, due to the cross‐linking of adsorbed water molecules.^[^ ^36^ ^]^ Meanwhile, nonmacrocyclic polysaccharides have flexible structures and are less affected by hydration because strong cross‐linking structures are not formed by water molecules. Therefore, either HSVM or a mortar and pestle was employed on a case‐by‐case basis to maintain the solid‐state information (properties) of guest molecules in aqueous solutions.

### 2.1 Water Solubilization of Hydrophobic Guest Molecules Using Polysaccharides

Shinkai and Sakurai et al. reported that schizophyllan, a type of β‐1,3‐glucan, can incorporate various guest molecules such as polyaniline,^[^ ^37^ ^]^ polythiophene,^[^ ^38^ ^]^ carbon nanotubes,^[^ ^39^, ^40^ ^]^ and porphyrins^[^ ^41^ ^]^ via the denaturation‐renaturation process. This process utilizes a property of schizophyllan, wherein its triple strands are denatured into single strands using dimethyl sulfoxide (DMSO) and renatured into triple strands after it is reintroduced in water. The encapsulation of guest molecules by schizophyllan is a crucial step during triplex refolding. However, potentially toxic DMSO used for denaturation remains in the aqueous solution. The existing water‐solubilization methods are not well‐suited for solid‐state guest molecules. These methods typically require the molecules to be pre‐dispersed in a basic aqueous solution or DMSO, which complicates their solubilization and makes it challenging to retain the original solid‐state information. We proposed HSVM for the solubilization of guest molecules using unmodified polysaccharides without requiring polar organic solvents such as DMSO (Scheme 2). This method has been used for complexing polysaccharides with fullerene derivatives (**1**–**5**),^[^ ^42^, ^43^ ^]^ porphyrin derivatives (**6**–**12**),^[^ ^35^, ^44^, ^45^, ^46^, ^47^ ^]^ porphyrin analogs (**13**–**17**),^[^ ^48^, ^49^ ^]^ anti‐inflammatory agents (**18**),^[^ ^50^ ^]^ carborane derivatives (**19**),^[^ ^51^ ^]^ triphenyl borane (**20**),^[^ ^52^ ^]^ tetraphenylethylene and its derivatives (**21**–**23**),^[^ ^53^, ^54^ ^]^ and 4,4′‐difluorobenzophenone (**24**),^[^ ^55^ ^]^ thereby enhancing their solubility (**Figure** **1**, **2**, **5**). When polysaccharides modified with a Protein A mimetic^[^ ^56^ ^]^ used as the conjugation unit with an antibody,^[^ ^51^ ^]^ hydroxypropyl groups^[^ ^57^ ^]^ used to impart thermoresponsiveness,^[^ ^58^ ^]^ and folic acid groups^[^ ^59^, ^60^ ^]^ used as a targeting molecule for selective tumor recognition^[^ ^61^ ^]^ were used in HSVM, guest molecules were efficiently incorporated without requiring the removal of substituents.

**Figure 3:**
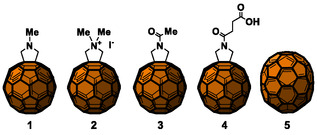
Chemical structures of fullerenes.

**Figure 4:**
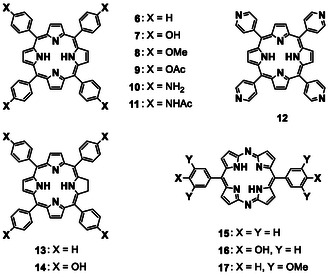
Chemical structures of porphyrin derivatives and analogs.

**Figure 5:**
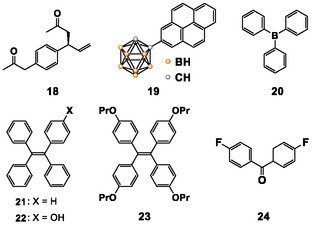
Chemical structures of the anti‐inflammatory agent, carborane derivative, tetraphenylethylene and its derivatives, and 4,4′‐difluorobenzophenone.

Fullerene derivatives **1** and **3**–**5** were solubilized in water using GL, with extraction rates of >64% (**Table** **1**, Figure 3). In contrast, **2** was scarcely solubilized, with an extraction rate of <5%. This was likely because the association constants of cyclodextrins with anionic porphyrin derivatives were considerably higher than those of cationic porphyrin derivatives^[^ ^62^ ^]^ due to electrostatic repulsion in the positively polarized cyclodextrin cavity resulting from the inductive effect of ether oxygen atoms.^[^ ^62^ ^]^ As cyclodextrin and GL containing the same glucose residues have similar cavities, it is not surprising that **2** did not form a complex with GL.

**Table 1:** Extraction rate of fullerene derivatives (**1–5**) via HSVM using polysaccharides.

Solubilizing agent	[SA] (g/L)a)	Guest molecule	[G] (mM)b)	Extraction rate (%)c)	Ref.
GL	2.00	**1**	0.30	75	[43]
GL	2.00	**2**	<0.32	<5	[43]
GL	2.00	**3**	0.32	80	[43]
GL	2.00	**4**	0.38	95	[43]
GL	2.70	**5**	0.27	64	[42]

a) SA denotes the solubilizing agent.

b) G denotes the guest molecule.

c) Extraction rates are determined as (G extracted in an aqueous solution)/(G used).

Porphyrin derivatives **6**–**8** achieved higher extraction rates with tamarind gum (TG) than with GL (**Table** **2**, Figure 1 and 4); however, porphyrin derivative **10** exhibited the opposite trend. **10** was further solubilized with λ‐carrageenan (CGN), pullulan (PL), arabinogalactan (AG), and sodium hyaluronate (SH) in water to investigate the relationship between the solubility of polysaccharides and persistence length (*l*_p_) with respect to the extraction rate of **10** (Figure 1 and 4). The chains of polysaccharides adsorbed on solid surfaces have different conformations: trains (chain segments in contact with the solid surface); loops (segments that connect the trains in a solution); and a tail (ends of the chains).^[^ ^63^, ^64^ ^]^ Thus, soft polysaccharides are more likely to interact with **10** by easily bending into loops. The *l*_p_ values of CGN, TG, PL, AG, and SH were 2.3–3.0, 6–8, <2, 6–9, and 4–6 nm, respectively,^[^ ^65^, ^66^, ^67^, ^68^, ^69^ ^]^ indicating poor correlation between the stiffness of polysaccharides and the solubility of **10**. Furthermore, the solubility of the complexes is not only determined by the water solubility of polysaccharides but also depends largely on the compatibility between the guest molecules and polysaccharides. Therefore, at present, the solubility of the complexes cannot be determined without actual experiments. Actually, compound **11** containing amide groups achieved an extremely low extraction rate compared with other porphyrin derivatives. Chlorins (Figure 4: **13** and **14**) could be extracted in water via the same approach as the porphyrin derivatives; however, the extraction rate of diazaporphyrins (**15**–**17**) was reduced. The reasons for these phenomena are not clear at present.

**Table 2:** Extraction rates of porphyrin derivatives and analogs (**6–17**) via HSVM using polysaccharides.

Solubilizing agent	[SA] (g/L)a)	Guest molecule	[G] (mM)b)	Extraction rate (%)c)	Ref.
GL	5.0	**6**	0.34	34	[35]
TG	5.0	**6**	0.45	45	[47]
GL	5.0	**7**	0.18	18	[35]
TG	5.0	**7**	0.45	45	[47]
HPCd)	5.0	**7**	0.03	3	[58]
GL	5.0	**8**	0.21	21	[35]
TG	5.0	**8**	0.66	66	[47]
TG	5.0	**9**	0.56	56	[47]
GL	5.0	**10**	0.78	78	[44]
TG	5.0	**10**	0.53	53	[47]
CGN	5.0	**10**	0.22	22	[35]
PL	5.0	**10**	0.64	64	[35]
AGe)	5.0	**10**	–	<2	[35]
HA	5.0	**10**	–	<2	[35]
TG	5.0	**11**	0.18	18	[47]
PL	6.7	**11**	0.18	14	[61]
FAPL‐1.8f)	6.7	**11**	0.16	12	[61]
FAPL‐2.8f)	6.7	**11**	0.11	8	[61]
FAPL‐5.4f)	6.7	**11**	0.10	8	[61]
GL	2.0	**12**	0.73	35	[35]
CGN	5.0	**13**	0.24	24	[48]
CGN	2.0	**14**	0.53	53	[48]
GL	3.3	**15**	0.32	19	[49]
GL	3.3	**16**	0.29	17	[49]
GL	3.3	**17**	0.31	19	[49]

a) SA denotes the solubilizing agent.

b) G denotes the guest molecule.

c) Extraction rates are determined as (G extracted in an aqueous solution)/(G used).

d) After the HSVM treatment, a complex of **7** with HPC was extracted using water at 55 °C.

e) AG denotes arabinogalactan.

f) Substitution degrees of folic acid in pullulan were determined to be 1.8, 2.8, and 5.4 per 100 glucose units.

Hydroxypropylcellulose (Figure 1: HPC)—a thermoresponsive polysaccharide—has a lower extraction rate for **7** compared with GL or TG; however, the HPC–**7** complex enables the release of **7** via a heating process that mimics its mitochondrial environment. Thus, **7** can be effectively used in mitochondria‐targeted photodynamic therapy.

PL modified by folic acid (FAPL) was synthesized using Steglich et al.'s method (Figure 1).^[^ ^70^ ^]^ FAPL‐1.8, FAPL‐2.8, and FAPL‐5.4 contained 1.8, 2.8, and 5.4 modified folic acid moieties per 100 glucose units. The concentrations of **11** in the aqueous solutions of PL–**11**, FAPL‐1.8–**11**, FAPL‐2.8–**11**, and FAPL‐5.4–**11** complexes were 0.18, 0.16, 0.11, and 0.10 mM, respectively (Table 2). FAPLs with a higher degree of folic acid modification could not efficiently solubilize **11**; thus, folic acid moieties did not act as hydrophobic sites as in the nanogels, but rather as steric hindrances in the hydrophobic field. Therefore, the existence of another uptake pathway was suggested herein. Contrarily, the photodynamic activity of the FAPL‐5.4–**11** complex was considerably higher than that of the PL–**11** complex. The FAPL‐5.4–**11** complex could more effectively inhibit tumor growth than the PL–**11** complex and Photofrin under light‐irradiation in vivo experiments. This was because of its enhanced intracellular uptakes induced by the folic acid moieties of FAPL as the uptake pathway was not merely mediated by Folr1.

Small guest molecules (**18**–**22**), besides **19**, could be solubilized in water with high extraction rates (**Table** **3**, Figure 5). The complexes of **23** and **24** with polysaccharides were prepared by grinding them in a mortar and pestle, which was a milder process than HSVM, to retain the solid‐state information of the guest molecules (Table 3). Although the extraction rates were slightly lower, these guest molecules were water‐soluble, similar to that observed in HSVM.

**Table 3:** Extraction rates of small guest molecules (**18–24**) via HSVM or mortar and pestle grinding using polysaccharides.

Solubilizing agent	[SA] (g/L)a)	Guest molecule	[G] (mM)b)	Extraction rate (%)c)	Ref.
GL	1.5	**18**	2.64	85	[35]
HA	5.0	**19**	0.73	37	[51]
TG	2.5	**20**	2.03	81	[52]
PL	5.0	**21**	1.90	76	[53]
CGN	5.0	**21**	2.43	97	[53]
PL	5.0	**22**	1.61	64	[53]
CGN	5.0	**22**	1.68	67	[53]
PLd)	5.0	**23**−CA	0.11–0.26e)	7–17	[54]
PL	5.0	**23**−CA	0.03–0.07e)	2–5	[54]
CGNd)	5.0	**24**	0.54	23	[55]

a) SA denotes the solubilizing agent.

b) G denotes the guest molecule.

c) Extraction rates are determined as (G extracted in an aqueous solution)/(G used).

d) These complexes are prepared by grinding in a mortar and pestle.

e) Variation in the concentrations of **23** depending on its final state in the complex.

### 2.2 Water Solubilization of Hydrophobic Guest Molecules Using Polypeptides

**Figure 6:**
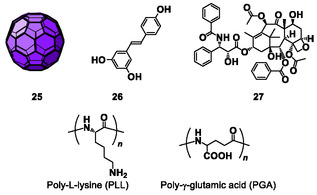
Chemical structures of guest molecules and polypeptides used as solubilizing agents.

Polypeptides can also be used as solubilizing agents in HSVM. They formed complexes with C_60_ (**25**),^[^ ^71^ ^]^ porphyrin derivatives (**6** and **7**),^[^ ^71^ ^]^ tetraphenylethylene (**21**),^[^ ^71^ ^]^ resveratrol (**26**),^[^ ^72^ ^]^ and paclitaxel (**27**)^[^ ^73^ ^]^ (**Figure** 2, 3, 6, **Table** **4**). Poly‐L‐lysine (PLL)–**7** and poly‐γ‐glutamic acid (PGA)–**7** complexes, similar to polysaccharide–**7** complexes, exhibited high photodynamic activities.^[^ ^15^, ^70^ ^]^ Treatment of a rat basophilic leukemia cell line (RBL‐2H3) with PLL–**26** and PGA–**26** complexes suppressed allergic responses in vitro.^[^ ^72^ ^]^ The complex of an anticancer drug, paclitaxel (**27**), with PLL effectively induced cancer cell death via apoptosis without causing any harmful side effects on the healthy cells.^[^ ^73^ ^]^ Although natural polymers are generally highly biocompatible, PLL is potentially cytotoxic due to its cationic character.^[^ ^74^ ^]^ By optimizing the molar ratio of interests using PLL for reducing the amount of free PLL in the aqueous solution of the PLL–**10** complex, its dark toxicity was considerably reduced than that of free PLL.^[^ ^75^ ^]^ This indicated that the complexed PLL exhibited almost no dark toxicity in the hydrophobic cavities and could be dissolved in water. In contrast, the PLL–**10** complex exhibited high photodynamic activity under light irradiation of 610–740 nm.

**Table 4:** Extraction rates of hydrophobic guest molecules via HSVM using polypeptides.

Solubilizing agent	[SA] (g/L)a)	Guest molecule	[G] (mM)b)	Extraction rate (%)c)	Ref.
PLL	5.0	**25**	1.90	76	[71]
PGA	5.0	**25**	0.44	18	[71]
PLL	5.0	**6**	1.00	100	[71]
PGA	5.0	**6**	0.65	65	[71]
PLL	5.0	**7**	0.20	20	[71]
PGA	5.0	**7**	0.58	58	[71]
PLL	5.0	**21**	1.30	52	[71]
PGA	5.0	**21**	0.64	26	[71]
PLL	5.0	**26**	0.96	16	[72]
PGA	5.0	**26**	1.56	26	[72]
PLL	5.0	**27**	0.08	8	[73]
Geld)	5.0	**27**	0.06	6	[73]

a) SA denotes the solubilizing agent.

b) G denotes the guest molecule.

c) Extraction rates are determined as (G extracted in an aqueous solution)/(G used).

d) Gel denotes gelatin.

## 3 Retention of Luminescence of Aggregation‐Induced Emission Luminogens in Aqueous Solutions

The hydrophobic guest molecules self‐aggregate within the natural polymer, forming nanoparticles with a size of ≈50–300 nm and broadening their absorption spectra. Therefore, many fluorescent materials, such as porphyrins, are self‐quenching. This phenomenon, called aggregation‐caused quenching (ACQ), is observed in many fluorescent materials and hinders the development of efficient sensors that can operate in water. To solve this problem, aggregation‐induced emission (AIE) without ACQ was proposed.^[^ ^76^, ^77^, ^78^, ^79^, ^80^, ^81^ ^]^ Typical AIE luminogens (AIEgens) such as tetraphenylethylene and hexaphenylsilole exhibit high fluorescence upon self‐aggregation. In solid states, AIEgens exhibit strong fluorescence due to restriction of their rotational and vibrational motions.^[^ ^76^, ^77^, ^78^, ^79^, ^80^, ^81^ ^]^ In particular, as the excited state of tetraphenylethylene derivatives is deactivated by the rotation of the central C=C double bond, as in the (*E*)–(*Z*) photoisomerization of stilbene, the suppression of the rotation of the C=C double bond in the solid state increases the fluorescence intensity.^[^ ^82^, ^83^, ^84^ ^]^

Tetraphenylethylene **21** and its derivative **22** were solubilized in water via HSVM using PL as the solubilizing agent.^[^ ^48^ ^]^ The aqueous solutions were fluorescent, as expected. Although tetraphenylethylene derivatives exhibit fluorescence in the γ‐cyclodextrin cavity in water, the emission is limited to short‐wavelength monomers.^[^ ^85^, ^86^ ^]^ Consequently, the cyclodextrin cavity restricts the rotation of the C=C double bond. This mechanism for restricting molecular motion differs from the self‐aggregation of compounds **21** and **22** by their formation of the PL complexes. The fluorescence images showed the intracellular uptake of the PL–**22** complex by HeLa cells. In contrast, the PL–**21** complex was directly adsorbed on the surface of a glass culture plate. These results are consistent with the finding that guest molecules with phenol unit(s) are readily taken up by HeLa cells.

## 4 Retention of the Crystal Chiral Information of Tetraphenylethylene in Aqueous Solutions

**Figure 7:**
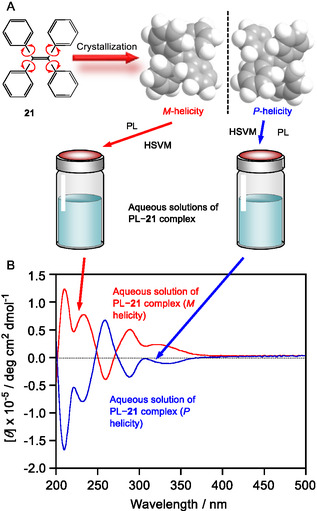
A) Preparation of aqueous solutions of PL–**21** complexes that incorporated *M*‐helical and *P*‐helical crystals of **21** via HSVM and B) CD spectra of the 1‐PL complexes prepared from *M*‐helical (red line) and *P*‐helical crystals (blue line; 1 mm pathlength cell, 25 °C, water). Reproduced with permission.^[^ ^53^ ^]^ Copyright 2020, Wiley‐VCH.

Soai et al. reported that **21** and their derivatives exhibited self‐aggregation‐induced circular dichroism (CD) signals and formed *P*‐helical and *M*‐helical crystals^[^ ^87^ ^]^ that were separated based on their crystal shapes. Therefore, this method was employed to separate *P*‐helical and *M*‐helical crystals. Then, each crystal was solubilized via HSVM using PL. The corresponding CD spectra were mirror images of the aqueous solutions of PL complexes prepared from *P*‐helical and *M*‐helical crystals, respectively, and were consistent with the helicity reported by Soai et al. (**Figure** **7**A). The separate peaks observed at 340, 288, 262, 231, and 209 nm in the CD spectra of *P*‐helical or *M*‐helical crystals **21** in KBr were correlated to the peaks observed at 335, 288, 259, 232, and 210 nm in the CD spectrum of the PL–**21** complex (Figure 7B).^[^ ^53^, ^88^ ^]^ Results revealed the initial helicity of crystal **21** remained in the complex with PL in water because the PL–**21** complex was prepared via HSVM, in which PL and guest molecule **21** are mixed in the solid state (Figure 7).

## 5 Aqueous Solutions with Polymorph Information on Aggregation‐Induced Emission Luminogen Solids

**Scheme 3:**
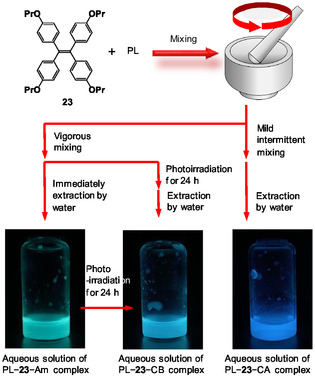
Preparation of aqueous solutions of PL–**23**–Am, PL–**23**–CB, and PL–**23**–CA complexes by grinding using a ceramic pestle and mortar and photoirradiation. Reproduced with permission.^[^ ^54^ ^]^ Copyright 2021, ACS.

Dong et al. reported that 1,1,2,2‐tetrakis(4‐propoxyphenyl)ethene (**23**), which is a tetraphenylethylene derivative, exhibited AIE and multicolored emission switching in its solid state.^[^ ^89^ ^]^ Crystal A of **23** (**23**–CA), prepared by recrystallization from the dichloromethane–methanol mixture, exhibited a deep‐blue emission at 448 nm when excited by light with a wavelength of 370 nm. After grinding, the crystal changed to amorphous powder (**23**–Am) with a green emission at 491 nm. As **23**–Am was in a metastable state, the amorphous powder transitioned into another crystal B (**23**–CB) with a sky‐blue emission at 462 nm. Thus, the solubility of solids can be decreased in water with polysaccharides while maintaining their information on solids, such as the asymmetry of **21**. However, when **23**–CA, **23**–CB, and **23**–Am were mixed with PL via HSVM to form complexes, only PL–**23**–Am complexes were formed in all cases.^[^ ^54^ ^]^ This was because the high pressure applied by the HSVM system transformed **23** into its amorphous form. Therefore, a hand grinder with a ceramic pestle and mortar was used—which is a milder process than HSVM—to mix **23**–CA and PL. However, with continued grinding, the entire solid became **23**–Am. The PL–**23**–Am mixture was extracted using water, and the aqueous solution of the PL–**23**–Am complex exhibited a green emission at 491 nm (**Scheme** **3**). In contrast, when solid or aqueous PL–**23**–Am mixture was placed under fluorescent light, it transformed into the PL–**23**–CB complex. An aqueous solution of the PL–**23**–CA complex with a deep‐blue emission at 448 nm was prepared (Scheme 3) by repeating the mild grinding of the **23**–CA and PL mixture for 15 min, followed by standing for 15 min and grinding for 1 h, for four times. The complex emitted green radiation immediately after mixing; however, after 15 min, the green emission completely returned to deep‐blue emission. This phenomenon was consistent with the result that the residual small crystals of **23**–CA acted as nuclei to transfer **23**–Am to **23**–CA.^[^ ^89^ ^]^ An aqueous solution of the PL–**23**–CA complex with a deep‐blue emission at 448 nm was obtained by intermittently mixing the solution. Although the components of complexes were the same, their aqueous solutions that retained the three fluorescent properties of solids were obtained.^[^ ^54^ ^]^

## 6 Phosphorescence in Aqueous Solution

Morantz et al. discovered that metal‐free organic materials exhibit room‐temperature phosphorescence (RTP).^[^ ^90^ ^]^ High‐performance organic materials showing RTP were obtained when heavy atoms (such as Br and I) and heteroatoms (such as O and S) were introduced into these organic materials, which facilitated crystalline packaging. The heavy atoms and heteroatoms suppressed the pathways of nonradiative relaxation and molecular motion, respectively.^[^ ^22^, ^91^ ^]^ However, in many single‐component systems, trace amounts of impurities can considerably affect athe RTP properties.^[^ ^92^ ^]^ RTP emissions can also be enhanced using host–guest complexes.^[^ ^93^, ^94^ ^]^ An and Huang et al. reported that organic RTP was solubilized in water by incorporation in an amphiphilic triblock copolymer and the complex showed long phosphorescence with an emission lifetime of 0.65 s.^[^ ^95^ ^]^

4,4′‐difluorobenzophenone (Figure 5: **24**) exhibited a longer phosphorescence lifetime and higher quantum yield compared with benzophenone.^[^ ^96^ ^]^ The CGN–**24** complex was synthesized via grinding using a ceramic pestle and mortar, as well as via HSVM. The high pressure from the HSVM system may have also driven the transformation of **24** into an amorphous form. The mean lifetime of the CGN–**24** complex prepared via grinding (<τ> = 29.7 μs) was longer than that prepared using HSVM (<τ> = 8.3 μs).^[^ ^54^ ^]^ However, the <τ> values of **24** in the complex state were considerably lower than those in the solid state (1297 μs).^[^ ^96^ ^]^ This confirmed the successful preparation of ASIS with crystallization‐induced RTP using the aqueous solution of CGN–**24** complexes.

## 7 Fluorescent Sensing of Small Aromatic Guest Molecules by Charge‐Transfer Complexes

**Figure 8:**
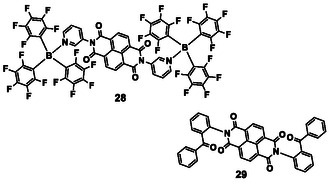
Chemical structures of NDI derivatives.

1,4,5,8‐Naphthalenetetracarboxydiimide (NDI) derivatives have strong fluorescence due to donor–acceptor interactions with small aromatic guest molecules.^[^ ^97^, ^98^, ^99^ ^]^ In addition, the fluorescence remains almost unchanged due to low association constants and the addition of small aromatic guest molecules in organic solvents. In contrast, in crystals, NDI derivatives can function as porous supramolecular host compounds to encapsulate aromatic guest molecules. Colquhoun et al. synthesized a cyclic NDI derivative^[^ ^100^, ^101^ ^]^ and Kitagawa et al.^[^ ^102^ ^]^ and Myers et al.^[^ ^103^ ^]^ independently used metal–organic frameworks containing NDI derivatives to incorporate guest molecules into the cavities of these materials to generate strong complexes. Hisaeda and Ono et al. synthesized NDI derivatives **28** and **29** (**Figure** **8**) with bulky substituents, and demonstrated that they formed charge‐transfer complexes with small aromatic guest molecules in 1:2 and 1:1 ratios, respectively.^[^ ^104^, ^105^, ^106^ ^]^ The crystals of **28** and **29** adsorbed vapors of small aromatic guest molecules and exhibited distinct fluorescence wavelengths and intensities depending on the guest molecule due to the formation of charge‐transfer complexes. We and Ono et al. reported that when a chloroform solution of **29** was dropped onto a filter paper, dried, and then exposed to vapors of small aromatic guest molecules, the fluorescence maxima of **28** and **29** were dependent on the ionization potential of the guest molecules.^[^ ^107^ ^]^ The adsorption weight of **29** was only 15 μg per one sheet of filter paper, and the fluorescence intensity of **29**–toluene‐adsorbed paper changed slightly after incubation for 15 days at around 20 °C. Consequently, the guest molecules incorporated into **29** scarcely evaporated, making **29** an excellent sensor.

### 7.1 Aqueous Solutions with Fluorescence Information of Charge‐Transfer Interactions in Porous Supramolecular Host Molecules

*N,N*′‐Dipyrid‐3‐yl‐1,4,5,8‐naphthalenediimide linked to two tris(pentafluorophenyl)borane (**28**) was dissolved in water via HSVM using PLL as the solubilizing agent.^[^ ^108^ ^]^ To prepare the PLL–**28** complex incorporating toluene, the aqueous solution of PLL–**28** complex was subjected to liquid–liquid extraction using toluene. However, the absorbance of **28** decreased in the aqueous phase but was observed in the toluene phase. Compound **28** released from PLL dissolved in the toluene phase because toluene is a good solvent for **28**.

**Scheme 4:**
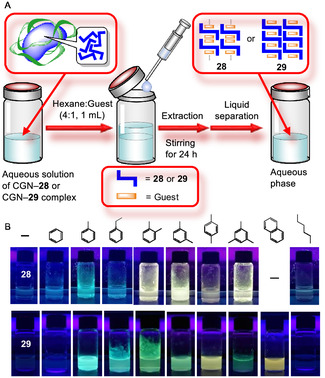
Synthesis of a complex of aromatic guest with biopolymer‐NDI derivatives complexes in aqueous media. A) Preparation of the complex of aromatic guest with CGN–**28** or **29**. B) Representative photograph of the fluorescence of the complex of aromatic guest with CGN–**28** or **29**. Reproduced with permission.^[^ ^108^ ^]^ Copyright 2022 and Reproduced with permission.^[^ ^109^ ^]^ Copyright 2024, Wiley‐VCH.

To suppress the release of **28** from PLL into toluene, hexane was added as a poor solvent in a toluene to hexane ratio of 1:4 v/v (**Scheme** **4**A). As a result, negligible amounts of **28** were generated in the toluene–hexane phase, with a strong fluorescence peak at 487 nm in the aqueous phase. The wavelengths of fluorescence peaks in the aqueous phase varied similarly depending on small aromatic guest molecules (Scheme 4B, **Table** **5**). Fluorescence intensities (*I*/*I*_0_) of the PLL–**28** complex^[^ ^108^ ^]^ were 2.3–7.6 and 1.7–10.0 times lower than those of **28** in the solid state (Table 5).^[^ ^105^ ^]^ Although the spectral trends of the complex were similar to those of small aromatic molecular complexes of **28** in the solid state, the long‐wavelength shifts of the PLL–**28** complex were small, except for benzene. These results indicated that a part of the supramolecular host **28** dissociated into *N,N*′‐dipyrid‐3‐yl‐1,4,5,8‐naphthalenediimide and tris(pentafluorophenyl)borane in water.

**Table 5:** Fluorescence maximum and intensity of the PLL–**28** complex and **28** in their solid states incorporating guest molecules (λ_ex_: 370 nm).

Guest molecules	PLL–**28** complex^[^ ^108^ ^]^		**28** a) in solid state^[^ ^106^ ^]^	
	λ_em_ (nm)	*I*/*I*_0_ b)	λ_em_ (nm)	*I*/*I*_0_ b)
Benzene	456	4.9	443	46
Toluene	487	7.6	488	76
Ethylbenzene	486	3.3	488	17
*o*‐Xylene	503	6.5	515	11
*m*‐Xylene	501	6.6	514	37
*p*‐Xylene	511	2.3	538	14
Mesitylene	514	3.2	533	6.2

a) Compound **28** in the amorphous state was exposed to the saturated vapor of each small aromatic guest molecule for 24 h at room temperature, after which the fluorescence spectrum was obtained.

b) Fluorescence response of **28** without and with the guest molecules (*I*/*I*_0_).

### 7.2 Aqueous Solutions with Fluorescence Information of Charge‐Transfer Interactions in Porous Host Molecules

We used **29** as the host molecule because it did not dissociate in water and contained two covalently linked bulky benzophenone groups.^[^ ^109^ ^]^ **29** was water‐solubilized using PLL in the same way as **28**, but it was barely soluble;^[^ ^108^ ^]^ therefore, the solubilizing agent was changed to CGN. Fluorescence intensities (*I*/*I*_0_) of the CGN–**29** complex were 6.7–19.4^[^ ^109^ ^]^ toward small aromatic guest molecules, except benzene; these values were considerably larger than those of the PLL–**28** complex (Scheme 4B, **Table** **6**)^[^ ^108^ ^]^ and 1.7 to 10.0 times smaller than those of **28** in the solid state.^[^ ^104^ ^]^ A comparison of the CGN–**29** complex and **29** in solid state with the same guest molecule showed that systems with larger long‐wavelength shifts exhibited larger *I*/*I*_0_ values.

**Table 6:** Fluorescence maximum and intensity of the CGN–**29** complex and **29** in their solid states incorporating guest molecules (λ_ex_: 370 nm).

Guest molecules	CGN–**29** complex^[^ ^109^ ^]^		**29** a) in solid state^[^ ^111^ ^]^	
	λ_em_ [nm]	*I*/*I*_0_ b)	λ_em_ [nm]	*I*/*I*_0_ b)
Benzene	450	3.8	443	**–** c)
Toluene	485	19.4	500	28
Ethylbenzene	486	12.5	477	**–** d)
*o*‐Xylene	497	16.2	479	7
*m*‐Xylene	497	15.8	488	4
*p*‐Xylene	511	6.7	521	40
Mesitylene	503	17.2	498	**–** d)

a) Compound **29** in an amorphous state was exposed to the saturated vapor of each small aromatic guest molecule for 24 h at room temperature, after which its fluorescence spectrum was obtained.

b) Fluorescence response of **29** without and with the guest molecules (*I*/*I*_0_).

c) There is an almost negligible response in the case of **29**–benzene complex.

d) No data in Ref. [108].

## 8 Conclusion and Outlooks

Herein, functional guest molecules were solubilized in water using natural polymers via HSVM; however, they self‐aggregated and caused self‐quenching of their photoexcited states. As a result, their fluorescence decreased and their ability to generate singlet oxygen considerably deteriorated. However, by complexing this self‐aggregated state with natural polymers, these functional guest molecules could be solubilized in water while retaining their solid‐state information. The solid‐state properties of functional guest molecules, such as chiral crystal formation, crystal polymorphism, mechanochromism, RTP, and fluorescence detection by molecular recognition, were retained in water as ASIS. These results will pave the way for new interdisciplinary research into supramolecular chemistry and organic crystallography, as well as facilitate maintaining a wider range of information in solids, even in water. For instance, crystal polymorphs could control drug release owing to their differing solubilities and stabilities, enabling their application in high‐precision drug‐delivery systems. Phosphorescent organic compounds could also be employed in bioimaging and phosphorescence‐based sensors. Concurrently, water‐soluble porous molecules might emerge as materials that function through host–guest recognition in water, with applications in sensors, catalytic reactions, the extraction of valuable metal ions, and the removal of environmental pollutants. We believe that these findings will greatly contribute to the advancement of functional materials for diverse fields.

## Conflict of Interest

The authors declare no conflict of interest.

## Author Contributions

**Keita Yamana**, **Riku Kawasaki**, and **Atsushi Ikeda** conducted a detailed literature review and drafted the manuscript. **Keita Yamana** and **Atsushi Ikeda** compiled the extraction rates of hydrophobic compounds from the data from each paper. **Keita Yamana** and **Riku Kawasaki** supervised the manuscript and provided critical feedback.

## Data Availability

The data that support the findings of this study are available from the corresponding author upon reasonable request.
